# Effect of Notch1 signaling on cellular proliferation and apoptosis in human laryngeal carcinoma

**DOI:** 10.1186/s12957-022-02728-6

**Published:** 2022-08-19

**Authors:** Dawei Li, Dan Xu, Yifei Zhang, Penghui Chen, Jin Xie

**Affiliations:** 1 Department of Otorhinolaryngology-Head & Neck Surgery, Xinhua Hospital, Shanghai Jiaotong University School of Medicine; Shanghai Jiaotong University School of Medicine Ear Institute; Shanghai Key Laboratory of Translational Medicine on Ear and Nose diseases, Shanghai, 200092 China; 2 Center for Translational Medicine, Yangpu Hospital, Tongji University School of Medicine, Shanghai, 200031 China

**Keywords:** Cell hypoxia, Notch1, Apoptosis, Cell proliferation, Laryngeal neoplasms

## Abstract

**Background:**

The occurrence and development of malignancies include excessive proliferation and apoptosis resistance in tumor cells. This study aimed to identify the effects of Notch1 signaling on proliferation and apoptosis of laryngeal cancer cells in a hypoxic microenvironment.

**Methods:**

Notch1 and Ki-67 expression in laryngeal squamous cell carcinoma (LSCC) tissues was detected by immunohistochemistry. The apoptotic index (AI) of LSCC was evaluated by the TUNEL method. Small interfering RNA (siRNA) was used to inhibit Notch1 expression in laryngeal cancer cells. Real-time PCR was used to measure Notch1, Hes1, and Hey1 mRNA expression, and Western blotting was used to measure Notch1 and Notch1 intracellular domain (N1ICD) protein expression. Annexin V-FITC/propidium iodide staining and Cell Counting Kit-8 assays were used to measure cell apoptosis and proliferation, respectively.

**Results:**

Notch1 expression was significantly related to the proliferation index (PI) and AI in LSCC tissues. Hypoxia could induce proliferation and inhibit apoptosis in cancer cells. Notch1 expression and Notch1 signaling activity could be upregulated by hypoxia. Suppressing *Notch1 signaling activity* in hypoxic cells could decrease proliferation and increase apoptosis.

**Conclusions:**

Our study has demonstrated that hypoxia may promote proliferation and inhibit apoptosis of laryngeal cancer cells. Notch1 signaling may play a pivotal role in regulating the proliferation and apoptosis resistance of laryngeal cancer cells under hypoxic conditions.

## Background

Laryngeal carcinoma has been considered as a common malignancy of the head and neck. Theoretically, the pathological processes of the occurrence and development of malignancies include excessive proliferation and apoptosis resistance of neoplastic cells. To date, the regulatory mechanism of the aberrant growth of laryngeal cancer cells has not been elucidated.

Hypoxia is a basic feature of the human solid tumor microenvironment. It is well known that hypoxia has a variety of effects on the regulation of apoptosis and proliferation in human neoplasms, which might be mediated by a set of regulatory mechanisms [1–3]. Previously, a number of studies have demonstrated that hypoxia could regulate multidrug resistance [4], stem-like biological properties [5], and metastasis [6] of laryngeal cancer cells. Unfortunately, the regulatory effect and mechanisms of hypoxia on proliferation and apoptosis of laryngeal cancer cells are still unclear.

Notch signaling is a highly conserved intercellular signaling pathway that can regulate different biological behaviors of neoplastic cells under hypoxic conditions and is mediated by regulating downstream target gene expression [7, 8]. Consistent with the conclusion of Dai et al. [9], our previous data showed that the expression level of Notch1 in laryngeal cancer tissues was significantly higher than that in normal mucosal tissues and was positively associated with lymph node metastasis and clinical stage [10], suggesting that aberrant Notch1 signaling may be involved in regulating the malignant process of laryngeal carcinoma. To date, studies have indicated that the effects of Notch1 signaling on proliferation and apoptosis of various neoplastic cells are still controversial [11–14]. Furthermore, Jiao et al. [15] confirmed that the overexpression of Notch1 in Hep-2 cells could suppress cellular proliferation and induce apoptosis. In contrast, Dai et al. [9] confirmed that Notch1 expression in Hep-2 cells could promote cell growth and inhibit apoptosis by regulating Notch1 target genes. As mentioned previously, the role of Notch1 signaling in regulating cellular proliferation and apoptosis in laryngeal carcinoma in Hep-2 cells in vitro is also controversial.

Accordingly, the purpose of the current study was to further examine the effects of Notch1 signaling on the regulation of apoptosis and proliferation of laryngeal cancer cells in a hypoxic microenvironment to clarify the regulatory role of Notch1 signaling in tumor progression.

## Methods

### Laryngeal cancer tissue samples

Specimens from 107 cases of laryngeal squamous cell carcinoma (LSCC) were collected from the Department of Otolaryngology, Head and Neck Surgery, Xinhua Hospital, Shanghai Jiaotong University School of Medicine between 1997 and 2020. Patients had been diagnosed with LSCC by histopathology. No patients included in this study had a history of radiotherapy or chemotherapy before surgery. The study was approved by the Ethics Committee of Xinhua Hospital (approval no. XHEC-D-2022-136). Written informed consent was obtained from the patients or their families.

### Immunohistochemistry

All tissue samples were obtained from the archives of the Department of Pathology. Immunostaining for Notch1 was performed on representative 5-μm-thick sections of LSCC tissue blocks. The following primary antibodies were incubated with the tissue sections overnight at 4 °C: rabbit anti-Notch1 monoclonal antibodies (Epitomics, Inc., Burlingame, CA, USA) (1:100 dilution) and rabbit anti-Ki-67 polyclonal antibodies (BA1508, Wuhan Boster, China) (1:800 dilution). The analysis was conducted by a two-step immunohistochemistry assay using a DAKO EnVision+ System (DAKO, Carpinteria, CA, USA).

### TUNEL assay

The apoptotic index (AI) was analyzed by TUNEL assays using an ApopTag Peroxidase In Situ Apoptosis Detection Kit (S7100, Chemicon International, Inc., USA). Tissue sections were deparaffinized and treated with proteinase K (20 μg/ml) for 15 min at room temperature. Then, the sections were quenched in 3.0% hydrogen peroxide in PBS for 5 min at room temperature, incubated with equilibration buffer for 1 min at room temperature, and incubated with TdT enzyme for 1 h at 37 °C. The sections were incubated with anti-digoxigenin conjugate for 30 min at room temperature, incubated with diaminobenzidine solution for 10 min at room temperature, and counterstained with hematoxylin.

### Evaluation of staining

All samples were independently reviewed by two senior pathologists with no prior knowledge of the patient information. Immunohistochemical staining of Notch1 was evaluated as described previously [16]. Furthermore, Notch1 protein expression was defined as negative (absent or weak immunostaining) or positive (moderate or strong immunostaining). Ki-67 immunostaining and the apoptosis rates of cancer cells were measured by the TUNEL method and obtained by reviewing a minimum of 1000 total cancer cells in the representative areas. The results are shown as the number of stained nuclei per 100 cells at 400× magnification.

### Laryngeal cancer cell lines

The AMC-HN-8 and Tu212 cell lines were obtained from the Institute of Biochemistry and Cell Biology, Shanghai Institute for Biological Sciences, Chinese Academy of Sciences. The cells were maintained in Dulbecco's modified Eagle's medium (DMEM; Gibco Corporation, USA) with 10% fetal bovine serum (HyClone, USA) and 1% penicillin/streptomycin (Invitrogen). For normoxic conditions, the cells were cultured in an incubator at 37 °C in a humidified atmosphere of 21% O_2_, 5% CO_2_, and 74% N_2_. For hypoxic conditions, the cells were placed in a hypoxic incubator (NuAire™ US Autoflow CO_2_ water-jacketed incubator) at 37 °C with 1% O_2_, 5% CO_2_, and 94% N_2_.

### Cell transfection

Double-stranded siRNA oligonucleotides targeting the Notch1 gene (Notch1-siRNA) (sense: 5′-CAGGGAGCAUGUGUAACAUTT-3′, antisense: 5′-AUGUUACACAUGCUCCCUGTT-3′) and the scrambled siRNA (sense: 5′-UUCUCCGAACGUGUCACGUTT-3′, antisense: 5′-ACGUGACACGUUCGGAGAATT-3′) were synthesized by Shanghai Genepharma Co. Ltd. (China). After 24 h of culture in antibiotic-free medium, Lipofectamine 2000 was used to transfect the siRNA (100 nM) into laryngeal cancer cells. After transfection for 24 h, the laryngeal cancer cells were collected for further analysis.

### Real-time PCR analysis

Total RNA was extracted from laryngeal cancer cells using TRIzol reagent (Invitrogen). According to the protocol of the reverse transcription kit, cDNA was reverse-transcribed from the isolated RNA. The primer sequences for PCR were as follows: Notch1 forward, 5′-CTACCTGTCAGACGTGGCCT-3′ and reverse, 5′-CGCAGAGGGTTGTATTGGTT-3′; Hes1 forward, 5′-TCTGAGCCAGCTGAAAACAC-3′ and reverse, 5′-GGTACTTCCCCAGCACACTT-3′; Hey1 forward, 5′-GGCTCCTTCCACTTACTGTCTC-3′ and reverse, 5′-ACTTTCCCCTCCCTCATTCTAC-3′; and GAPDH (internal control) forward, 5′-CATCTTCCAGGAGCGAGA-3′ and reverse, 5′-TGTTGTCATACTTCTCAT-3′. As performed in our previous study [4], real-time PCR was performed with a SYBR Green PCR kit (Takara Biotechnology Co., Ltd., Dalian, China) to measure the mRNA expression of Notch1, Hes1, Hey1, and GAPDH. The data were analyzed according to the 2^−△△CT^ method [17].

### Western blot analysis

Total protein was extracted from laryngeal cancer cells using RIPA lysis buffer. Then, total protein extracts were subjected to SDS–PAGE (5% stacking gel and 8% separating gel), transferred to PVDF membranes (Millipore), and blocked with 5% skimmed milk at room temperature for 2 h. Next, the membranes were immunoblotted with primary antibodies (Notch1 1:1000, rabbit anti-human; N1ICD 1:1000, rabbit anti-human; GAPDH, 1:1000, mouse anti-human) overnight at 4 °C and incubated with secondary antibodies (1:5000; room temperature, 1 h). The proteins were visualized and quantified by electrogenerated chemiluminescence.

### Cell proliferation assay

For the proliferation assay, AMC-HN-8 and Tu212 cells were plated in 96-well culture plates at a density of 5 × 10^3^ cells/well. After 12 h of incubation, the medium was replaced with new medium, and the cells were then incubated under normoxic or hypoxic conditions for another 48 h. Ten microliters of CCK-8 solution was added to each well, the cells were further cultured for 2 h, and the reaction was stopped. The optical density (OD) was measured at 450 nm on a microplate reader (Bio-Rad Laboratories, Inc.).

### Cell apoptosis analysis

The apoptosis index of neoplastic cells was evaluated by a BD FACSCalibur and an Annexin V-FITC Apoptosis Detection Kit (Bender Medsystems Inc. USA) according to the manufacturer’s protocol. Cells were cultured in 6-well plates (4 × 10^5^ cells/well) overnight at 37 °C. Then, after replacing the medium, the cells were cultured in hypoxic or normoxic conditions for 48 h. Next, neoplastic cells were washed in DMEM and resuspended in 190 μL of Tris-HCl buffer. Furthermore, 5 μL of Annexin-V-FITC and 5 μl of propidium iodide were added to the cell suspensions. The fluorescence intensity of the stained cells was assessed by flow cytometry (FCM).

### Statistical analysis

Continuous data were analyzed with Student’s *t*-test or one-way ANOVA. The correlation between ordinal variables was assessed by the Spearman’s rank correlation test. Statistical analyses were processed with SPSS 20.0 software. *P* < 0.05 was regarded as statistically significant.

## Results

### Immunoexpression of Notch1 in human LSCC tissues

**Fig. 1:**
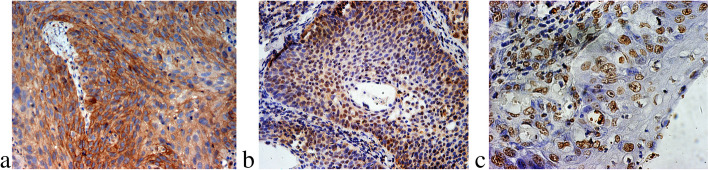
Immunohistochemical analysis of Notch1-, Ki-67-, and TUNEL-positive cells in LSCC tissues. **a** Notch1 was observed in the cell membranes and cytoplasm of cancer cells (×400). **b** Ki-67 was found in the nuclei of neoplastic cells (×400). **c** Apoptotic cells were assessed by the TUNEL method (×400)

LSCC samples from 107 laryngeal cancer patients were labeled for Notch1 by using immunohistochemistry. Immunostaining for Notch1 was observed in 68 (63.55%) of 107 laryngeal cancer tissue samples and was located in the membrane and cytoplasm of tumor cells (Fig. 1a).

### Measurement of apoptotic index and proliferative index in LSCC tissues and their relation to Notch1 expression

The proliferation level (Ki-67 expression) and apoptosis index (TUNEL staining) (Fig. 1 b and c) were determined to examine the staining of cell nuclei. The average proliferative and apoptotic indices (PI and AI) in human LSCC tissues were 27.80 ± 3.82% and 1.47 ± 0.18%, respectively. In the current study, the PI was obviously higher in the Notch1-positive group than in the Notch1-negative group (*P* < 0.05) (Table 1). On the other hand, the AI was significantly lower in the Notch1-positive group than in the Notch1-negative group (*P* < 0.05) (Table 1). Moreover, in LSCC tissues, Notch1 expression was positively correlated with the PI (*r*_s_ = 0.638, *P* < 0.05). Conversely, the expression of Notch1 was negatively correlated with the AI (*r*_s_ = −0.582, *P* < 0.05).

**Table 1:** Relationship between proliferative and apoptotic indices based on Notch1 expression in laryngeal squamous cell carcinoma

Indices (% ± SD)	Notch1 expression		
	Positive	Negative	*P*
Mean apoptotic index	1.31 ± 0.16%	1.75 ± 0.21%	< 0.05
Mean proliferative index	30.48 ± 4.16%	23.12 ± 3.02%	< 0.05

### Hypoxia promoted proliferation and inhibited apoptosis of laryngeal cancer cells

**Fig. 2:**
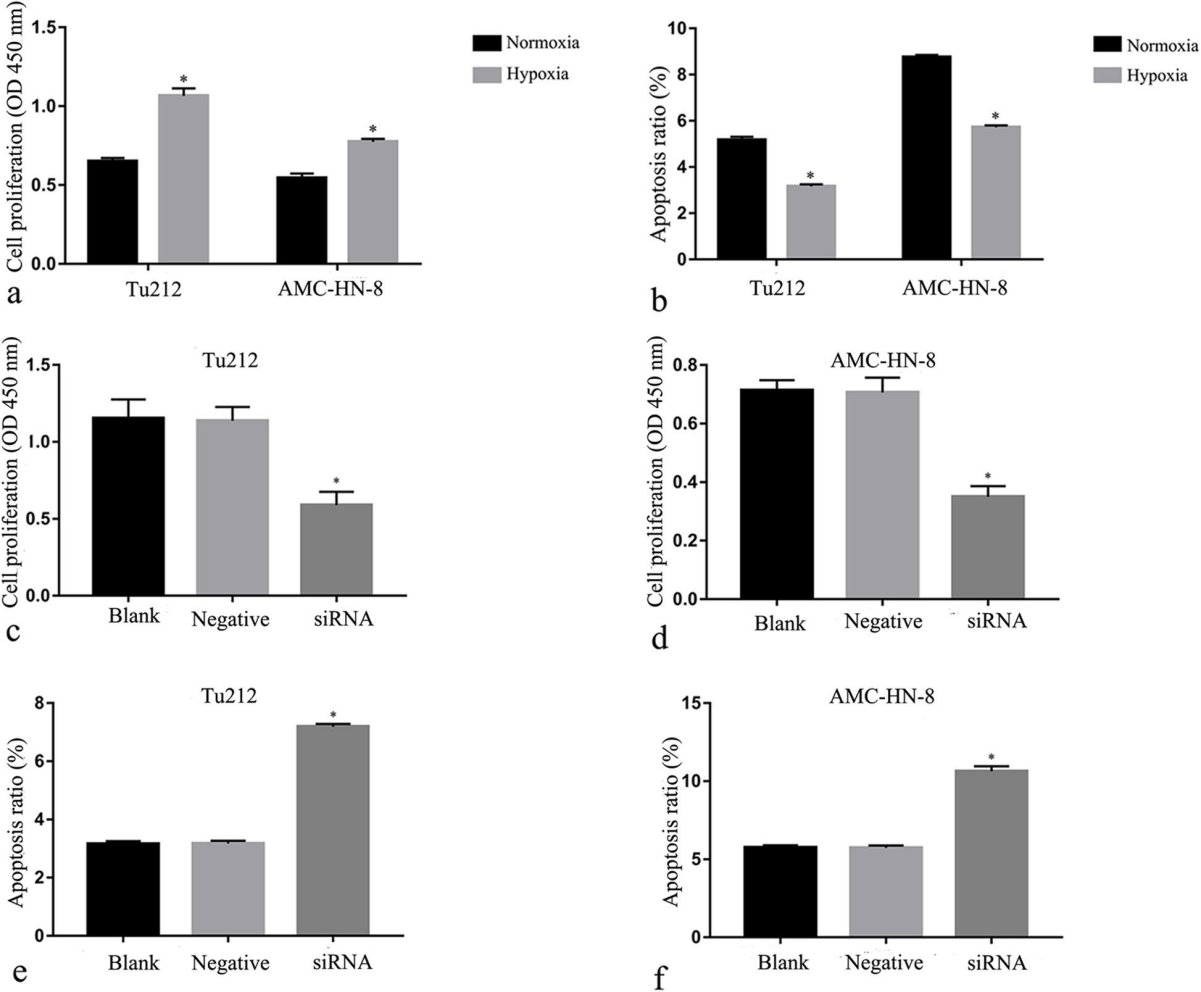
Changes in proliferation and apoptosis of laryngeal carcinoma cells. Hypoxia promoted proliferation (**a**) and suppressed apoptosis (**b**) of cancer cells. Suppressing Notch1 signaling activity reduced the proliferation of TU212 (**c**) and AMC-HN-8 (**d**) cells under hypoxia; conversely, it increased apoptosis in TU212 (**e**) and AMC-HN-8 (**f**) cells under hypoxia. *P < 0.05 versus control groups

AMC-HN-8 and Tu212 cells were incubated in normoxic or hypoxic conditions for 48 h to observe the effects of hypoxia on proliferation and apoptosis in neoplastic cells. Apoptosis and proliferation in laryngeal cancer cells were examined by Annexin V-FITC/propidium iodide staining and CCK-8 assays, respectively. As shown in Fig. 2a, the proliferation of neoplastic cells was significantly increased by hypoxia. On the other hand, the percent of apoptotic cells was decreased after incubation in hypoxic conditions for 48 h (Fig. 2b, *P* < 0.05).

### Hypoxia enhanced Notch1 expression and Notch1 signaling activity in laryngeal cancer cells

**Fig. 3:**
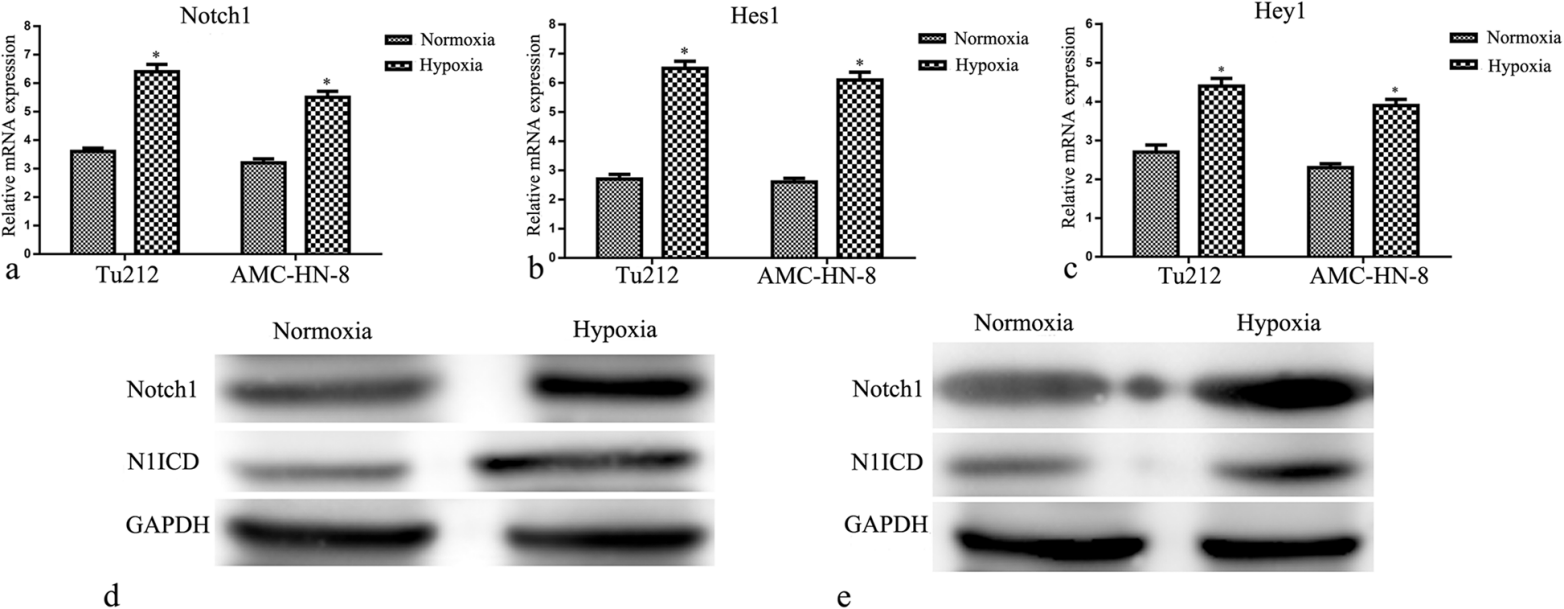
Effects of hypoxia on Notch1 expression and Notch1 signaling activity in laryngeal carcinoma cells. Real-time PCR estimated the expression of Notch1 (**a**), Hes1 (**b**), and Hey1 (**c**) mRNA in laryngeal cancer cells. Western blot evaluated the expression of Notch1 and N1ICD protein in Hep-2 (**d**) and AMC-HN-8 (**e**) cells. *P < 0.05 versus control groups

AMC-HN-8 and Tu212 cells *were incubated under normoxic or hypoxic conditions for 48 h*. *The real-time PC*R results *confirmed that hypoxia* significantly upregulated Notch1*, Hes1, and Hey1 mRNA expression in* tumor cells (*P* < 0.05) (Fig. 3 a–c)*. The Western blot results demonstrated that the protein expression of Notch1 and N1ICD in laryngeal cancer cells was enhanced by hypoxia* (*P* < 0.05) (Fig. 3 d and e)*. These results revealed that Notch1 expression and Notch1 signaling activity in laryngeal cancer cells could be enhanced by hypoxia.*

### Inhibiting Notch1 expression reduced the activity of Notch1 signaling in laryngeal cancer cells under hypoxic conditions

**Fig. 4:**
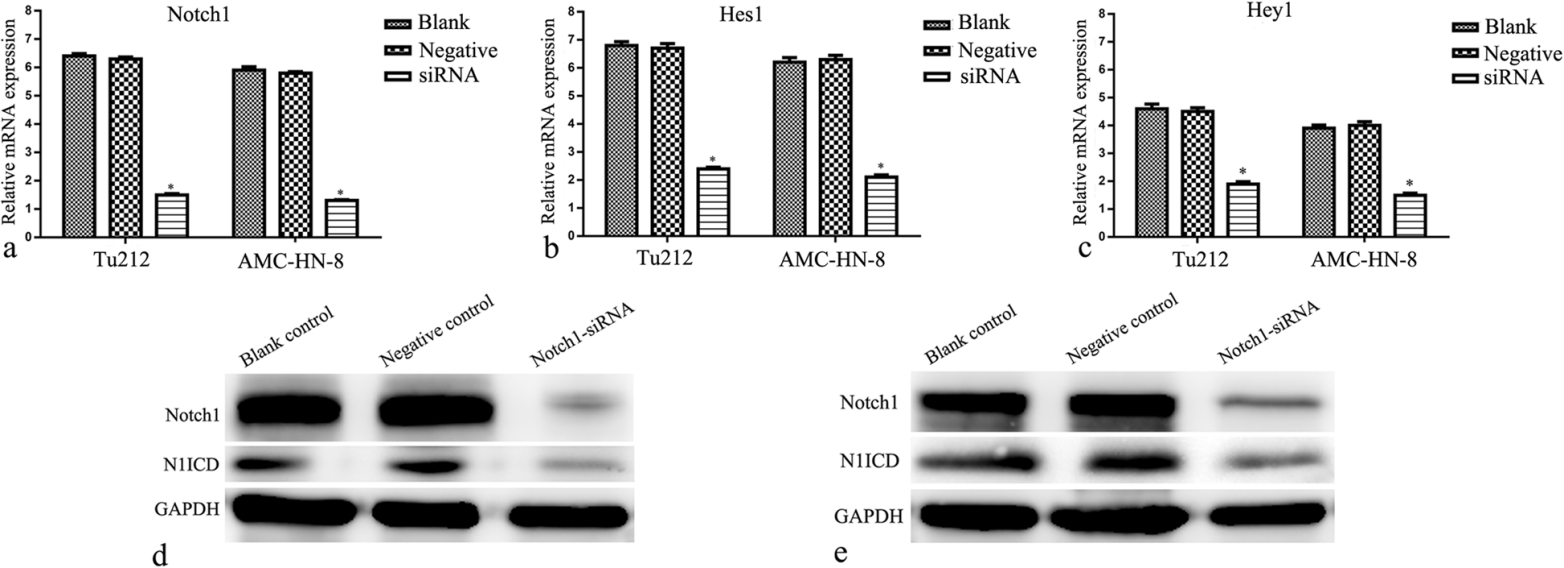
Effects of Notch1-siRNA on Notch1 signaling activity in hypoxic laryngeal carcinoma cells. Real-time PCR assessed the expression of Notch1 (**a**), Hes1 (**b**), and Hey1 (**c**) mRNA in Hep-2 and AMC-HN-8 cells under hypoxia. Western blot estimated Notch1 and N1ICD expression in Hep-2 (**d**) and AMC-HN-8 (**e**) cells under hypoxia. *P < 0.05 versus control groups

The real-time PCR results showed that Notch1, Hes1, and Hey1 mRNA levels in the Notch1-siRNA group were significantly lower than those in the control groups (*P* < 0.05) (Fig. 4 a–c). Likewise, the Western blot results confirmed that the protein expression levels of N1ICD and *Notch1* in the Notch1-siRNA group were lower than those in the control groups (*P* < 0.05) (Fig. 4 d and e). *These results indicated th*at *Notch1 signaling activity in hypoxic* laryngeal cancer cells cou*ld* be downregulated by *inhibiting* Notch1 expression.

### Inhibiting Notch1 signaling decreased proliferation and induced apoptosis in hypoxic laryngeal carcinoma cells

To examine the effects of *Notch1 signaling* on cell proliferation and apoptosis in hypoxia, AMC-HN-8 and Tu212 cells were evaluated by using the CCK-8 method and Annexin V-FITC/propidium iodide staining. *As* shown in Fig. 2 c–f, suppressing *the activity of Notch1 signaling* in AMC-HN-8 and Tu212 cells by transfection with siRNA evidently decreased cell proliferation and increased the apoptosis rate (both *P* < 0.05).

## Discussion

To our knowledge, abnormal proliferation and apoptosis of neoplastic cells always underlie the pathological process of carcinogenesis and progression in various malignancies [18]. However, the regulatory mechanisms related to the apoptosis and proliferation of human neoplasm cells still remain unclear. Thus, it is significant to explore the regulatory mechanisms of the disturbance between cell apoptosis and proliferation in human laryngeal carcinoma.

Hypoxia, which is an essential feature of the tumor microenvironment, induces a set of functionally adaptive responses of neoplastic cells, including cell proliferation and apoptosis [1–3], which are mediated by a series of molecular mechanisms. Likewise, a number of studies have demonstrated that hypoxia regulates a variety of malignant biological phenotypes of laryngeal carcinoma cells [4–6]. To date, there have been few reports on the regulatory effect of the hypoxic microenvironment on proliferation and apoptosis of laryngeal cancer cells. In this study, we examined the proliferation and apoptosis of AMC-HN-8 and Tu212 cells under normoxic and hypoxic conditions. Consequently, the proliferation of AMC-HN-8 and Tu212 cells was significantly increased by hypoxia. Conversely, apoptosis of AMC-HN-8 and Tu212 cells was decreased by hypoxia. These results suggest that hypoxia can significantly enhance the proliferation and apoptosis resistance of laryngeal carcinoma cells.

Notch signaling, which is a core molecular signaling pathway, plays a vital role in regulating the expression of downstream target genes under hypoxic conditions, and these genes regulate a set of biological phenotypes in human neoplasms [7, 8]. Previously, Dai et al. [9] and our group [10] showed that Notch1 expression was upregulated in human LSCC tissues and was related to tumorigenesis and lymph node metastasis. Furthermore, we examined whether hypoxia was involved in regulating the activity of Notch1 signaling in laryngeal cancer cells, and the present study demonstrated that Notch1 expression and Notch1 signaling activity in Tu212 and AMC-HN-8 cells could be enhanced by hypoxia. These data revealed that in the hypoxic microenvironment, Notch1 signaling could regulate the malignant biological behaviors of laryngeal cancer cells.

To date, the effects of Notch1 signaling on cell apoptosis and proliferation in different kinds of human neoplasms are variable. Several studies have indicated that the Notch-1 pathway could promote cell proliferation and inhibit apoptosis in human pancreatic [11], gastric [12], and tongue cancer [19]. On the other hand, some authors have demonstrated that the Notch-1 pathway could suppress cell proliferation and induce apoptosis in human lung carcinoma [13] and esophageal squamous cell carcinoma [20]. The inconsistencies in research conclusions may be attributed to the regulatory heterogeneity among different types of tumor cells or tissues. Moreover, the study of Jiao et al. [15] has indicated that Notch1 signaling could inhibit cell proliferation and induce apoptosis of Hep-2 cells. In contrast, an in vitro study by Dai et al. [9] has demonstrated that Notch1 signaling could promote proliferation and inhibit apoptosis of Hep-2 cells. The conclusions of these two studies on laryngeal cancer cells are also controversial. In our in vitro study, inhibiting the activity of Notch1 signaling in Tu212 and AMC-HN-8 cells under hypoxic conditions induced cell apoptosis and reduced cell proliferation, which was consistent with the conclusions of Dai et al. [9]. Furthermore, our study indicated that Notch1 expression in laryngeal carcinoma tissues was negatively correlated with the AI and positively correlated with the PI, which further validates the conclusion of our in vitro study. Thus, Notch1 signaling may contribute to the proliferation and apoptotic resistance of laryngeal cancer cells in a hypoxic microenvironment.

## Conclusions

In summary, current research demonstrates that Notch1 signaling may play a critical role in regulating proliferation and apoptosis of laryngeal cancer cells in the hypoxic microenvironment and may be an effective target for the treatment of laryngeal cancer. Further research is needed to examine the molecular mechanisms by which Notch1 signaling regulates proliferation and apoptosis of human laryngeal carcinoma cells.

## Data Availability

The dataset supporting the conclusions of this article is included within the article.

## Abbreviations

LSCC: Laryngeal squamous cell carcinoma
AI: Apoptotic index
SiRNA: Small interfering RNA
N1ICD: Notch1 intracellular domain
PI: Proliferation index
Notch1-siRNA: Double-stranded siRNA oligonucleotide targeting Notch1 gene
CCK-8: Cell Counting Kit-8
TUNEL: TdT-mediated dUTP nick-end labeling
